# Antismoking messages and current cigarette smoking status in Somaliland: results from the Global Youth Tobacco Survey 2004

**DOI:** 10.1186/1752-1505-2-6

**Published:** 2008-05-23

**Authors:** Seter Siziya, Emmanuel Rudatsikira, Adamson S Muula

**Affiliations:** 1 Department of Community Medicine, University of Zambia, School of Medicine, Lusaka, Zambia; 2 Departments of Global Health, Epidemiology and Biostatistics, School of Public Health, Loma Linda University, California, USA; 3 Department of Community Health, University of Malawi, College of Medicine, Blantyre, Malawi

## Abstract

**Background:**

Tobacco is a leading cause of death globally. There are limited reports on current cigarette smoking prevalence and its associated-antismoking messages among adolescents in conflict zones of the world. We, therefore, conducted secondary analysis of data to estimate the prevalence of current cigarette smoking, and to determine associations of antismoking messages with smoking status.

**Methods:**

We used data from the Somaliland Global Youth Tobacco Survey (GYTS) of 2004 to estimate the prevalence of smoking. We also assessed whether being exposed to anti-smoking media, education and having discussed with family members on the harmful effects of smoking were associated with smoking. Logistic regression analysis was used to assess these associations. Current smoking was defined as having reported smoking cigarettes, even a single puff, in the last 30 days preceding the survey (main outcome).

**Results:**

Altogether 1563 adolescents participated in the survey. However, 1122 had data on the main outcome. Altogether, 15.8% of the respondents reported having smoked cigarettes (10.3% among males, and 11.1% among females). Factors that were associated with reported non-smoking were: discussing harmful effects of smoking cigarettes with their family members (OR = 0.61, 95% CI 0.52, 0.71); being taught that smoking makes teeth yellow, causes wrinkles and smokers smell badly (OR = 0.62, 95% CI 0.52, 0.74); being taught that people of the respondent's age do not smoke (OR = 0.81, 95% CI 0.69, 0.95); and having reported that religious organizations discouraged young people smoking (OR = 0.70, 95% CI 0.60, 0.82). However, exposure to a lot many antismoking messages at social gatherings was associated with smoking. Exposure to antismoking print media was not associated with smoking status.

**Conclusion:**

A combination of school and home based antismoking interventions may be effective in controlling adolescent smoking in Somaliland.

## Background

Tobacco use is a leading cause of morbidity and mortality globally. Tobacco's contribution to ill health is projected to rise if current smoking trends are not reduced across the world [1]. Although much of the long-term effects of smoking are observed in adulthood, the majority of smokers initiate the habit of smoking as adolescents.

There is a growing body of literature on adolescent tobacco use. Much of the research on the estimate of the prevalence and associated factors of adolescent smoking has been driven by the Global Youth Tobacco Survey (GYTS); an initiative spearheaded by the United States' Centers for Diseases Control and Prevention (CDC), the World Health Organization (WHO) and the Canadian Public Health Association [2], and a group of authors who have been conducting secondary analysis of GYTS data [3]. Traditional reporting of results from the GYTS has concentrated on ages 13 to 15 years, and estimates of prevalence and the general socio-environment that may be associated with teenage smoking.

Odenwald et al [4] have reported on the more than usual use of *khat *in Somalia as an indication of heightened prevalence of substance abuse in an environment of civil strife as is characteristic of most of Somalia. The prevalence of any tobacco use was 32.8% (26.8% among males, and 33.1% among females) and 15.8% currently smoked cigarettes (10.2% among males, and 11.1% among females) in the GYTS 2004 among adolescents 13 to 15 years of age [5].

While the prevalence of cigarette smoking in Somaliland has been reported, we are unaware of efforts to associate antismoking messages with current smoking status, and attempt to explain the prevalence of cigarette smoking within a conceptual or theoretical framework of health behaviors. We therefore carried out this study to estimate the prevalence of current cigarette smoking among all study participants in the Somaliland GYTS, identify positive antismoking messages, and explain their associations with current smoking status within theory-based outlook.

## Methods

### Study setting

The Republic of Somaliland is what was previously known as the Somaliland Protectorate under the British rule from 1884 to 1969. The country attained independence in June 1960 around the same time that Italian Somalia also achieved independence from Italy. On July 1st 1960 Somaliland joined the former Italian Somalia to form the Somali Republic. A civil war was fought from 1980 onwards resulting in the eventual collapse of the Somali Republic. Somaliland shares borders with Republic of Djibouti to the west, Federal Republic of Ethiopia to the south and, the Red Sea to the North and Somalia to the east.

### Study design and study participant recruitment

This is a secondary analysis of data from the Somaliland GYTS conducted in 2004. The Somaliland GYTS was a school-based survey of students in public primary classes 6 to 8, and Secondary 1. These classes were selected because they contained the majority of 13 to 15 years old adolescents in Somaliland. A two-stage cluster sample design was used to produce representative data for all of Somaliland. At the first stage, schools were selected with probability proportional to enrollment size. At the second stage, classes were randomly selected and all students in selected classes were eligible to participate. The overall response rate was 82.9%; the school response rate was 88%, the student response rate was 94.2%. Altogether 1563 students participated in the Somaliland GYTS.

### Questionnaire administration

A GYTS questionnaire was administered to students in their classrooms during one subject session. Only students who were present on the day a particular school was visited participated in the study. Students who were eligible but absent were not followed up. Questionnaires were self-completed on computer scan able survey sheets.

Students were asked among others the following questions: During the past 30 days (one month), on how many days did you smoke cigarettes? Has anyone in your family discussed the harmful effects of smoking with you? During the past 30 days (one month), how many anti-smoking media messages (e.g., television, radio, billboards, posters, newspapers, magazines, movies) have you seen or heard? When you go to sports events, fairs, concerts, community events, or social gatherings, how often do you see anti-smoking messages? During this school year, were you taught in any of your classes about the effects of smoking like it makes your teeth yellow, causes wrinkles, or makes you smell bad?; During the past year, did any health professionals explain to you why smoking is dangerous to your health? During the past year, did any religious organization discourage young people of your age from smoking? Responses to the questions were all pre-coded. A comprehensive description of the GYTS methodology has been reported elsewhere [6-8].

### Data analysis

We obtained the data set from the CDC. Data were analyzed in SPSS 14.0 (Chicago, IL, United States of America). Current cigarette smoking was defined as having smoked a cigarette, even a single puff, within the last 30 days prior to the survey. A weighted analysis was conducted to obtain the prevalence of current cigarette smoking as well as other relevant characteristics. Associations between current smoking and categorical variables forming tables with two rows and two columns were assessed using the Yates corrected Chi-square test, and the Pearson Chi-square test was used for higher contingency tables. Age and sex adjusted odds ratios of current smoking and 95%CI were also obtained in a multivariate logistic regression analysis. A cut off point for statistical significance was set at the 5% level.

In order to understand and explain the results of the quantitative analysis in this study, three health behavior theories i.e. the Health Belief Model (HBM), the Knowledge, Attitude and Practice (KAP) model and the social cognitive theory (SCT) were used to explained the observed effect estimates with relation to explanatory variables [9-13]. The SCT suggests that people's acquisition and maintenance of behavioral patterns is dependent on their own cognitive capacity, as well as on social-physical environment in which they are situated. The HBM on the other hand, promotes four constructs representing the perceived threat and net benefits: perceived susceptibility, perceived severity, perceived benefits, and perceived barriers. These concepts were proposed as accounting for people's "readiness to act." Other aspects of the model such as "cues to action," and efficacy, were added later to the model. Cues to action would activate readiness and stimulate overt behavior. In the KAP model, it is postulated that increased knowledge spurs people into changing their attitudes and eventually their behaviors. How knowledge specifically leads to behavioral change is not well explained.

## Results

Altogether 1563 adolescents participated in the survey. For 1285 study participants who reported their sex, 63.3% and 36.7% were males and females, respectively. However, 1122 responded to the question on whether they smoked cigarettes in the previous 30 days. Altogether 15.8% of the respondents reported having smoked cigarettes in the previous 30 days. There was no significant difference in the rate of reported smoking between sexes (10.3% in males versus 11.1% in females).

Males tended to be older than females. Female respondents were more exposed than males to antismoking media messages; antismoking messages at sports events, fairs, concerts, community events or social gathering; teaching on the dangers of smoking; and antismoking messages in newspapers or magazines. No associations were observed between being exposed to religious organization that discouraged young people from smoking and gender. Further description of the study population is presented in Table 1.

**Table 1:** Characteristics of the study population stratified by sex in the Somaliland Global Youth Tobacco Survey 2004

	Total	Males	Females	
Factor	n * (%)**	n * (%)**	n * (%)**	p value
Age				
≤ 11	347 (22.9)	211 (26.6)	66 (14.1)	<0.001
12	307 (20.2)	112 (13.8)	144 (31.2)	
13	129 (8.6)	55 (6.9)	31 (6.6)	
14	103 (6.9)	37 (4.7)	40 (8.9)	
15	142 (9.8)	69 (9.2)	41 (9.5)	
16	192 (13.2)	125 (16.5)	54 (12.1)	
17+	259 (18.2)	167 (22.4)	76 (17.7)	
Sex				
Male	813 (63.3)	-	-	-
Female	472 (36.7)	-	-	-
Frequency of antismoking media messages seen or heard				
A lot	835 (59.6)	468 (61.1)	288 (64.9)	<0.001
A few	338 (25.1)	186 (25.8)	94 (21.7)	
None	210 (15.2)	100 (13.2)	59 (13.4)	
Family member discussed harmful effects of smoking				
Yes	1045 (76.4)	615 (81.7)	309 (70.6)	<0.001
No	327 (23.6)	140 (18.3)	130 (29.4)	
Frequency of antismoking messages seen at sports events, fairs, concerts, community events or social gathering				
A lot	490 (34.5)	266 (34.1)	163 (37.5)	<0.001
Some	281 (19.8)	158 (20.5)	68 (15.6)	
None	655 (35.8)	354 (45.3)	205 (46.8)	
Taught dangers of smoking				
Yes	713 (50.9)	404 (52.4)	249 (55.3)	0.008
No	660 (49.1)	351 (47.6)	193 (44.7)	
Taught that smoking makes teeth yellow, causes wrinkles and smokers smell badly				
Yes	738 (52.0)	478 (60.6)	179 (39.5)	<0.001
No	657 (48.0)	298 (39.4)	264 (60.6)	
Frequency of antismoking posters seen				
A lot	566 (40.5)	314 (41.7)	160 (36.4)	<0.001
Some	339 (24.2)	173 (23.0)	111 (25.3)	
None	490 (35.3)	266 (35.4)	166 (38.3)	
Frequency of antismoking messages seen on billboards				
A lot	641 (45.2)	389 (50.3)	167 (37.1)	<0.001
Some	352 (24.7)	166 (21.5)	133 (29.2)	
None	419 (30.1)	216 (28.2)	148 (33.7)	
Frequency of antismoking messages seen in newspapers or magazines				
A lot	516 (36.9)	270 (35.7)	179 (39.9)	<0.001
Some	500 (36.7)	282 (37.9)	147 (34.3)	
None	372 (26.4)	204 (26.4)	117 (25.9)	
Taught that most people of respondent's age do not smoke cigarettes				
Yes	630 (46.3)	367 (49.5)	178 (41.7)	<0.001
No	753 (53.7)	391 (50.5)	255 (58.3)	
Health professional explained why smoking is dangerous to health				
Yes	602 (46.7)	352 (49.9)	177 (41.5)	<0.001
No	674 (53.3)	348 (50.1)	241 (58.5)	
Religious organization discouraged young people from smoking				
Yes	990 (73.9)	540 (74.7)	322 (74.9)	0.878
No	358 (26.1)	189 (25.3)	110 (25.1)	
Youth groups discouraged young people from smoking				
Yes	748 (58.9)	453 (62.2)	211 (53.6)	<0.001
No	513 (41.1)	272 (37.8)	177 (46.4)	
Current smoker				
Yes	183 (15.8)	65 (10.3)	37 (11.1)	0.248
No	939 (84.2)	554 (89.7)	283 (88.9)	

Numbers not adding up because of missing information.

* unweighted frequencies.

** weighted percentages.

Table 2 shows results of multivariate analysis after adjusting for age and sex. The following factors were not associated with reported smoking: being taught dangers of smoking, frequency of antismoking posters seen, frequency of antismoking messages seen in newspapers or magazines, youth groups discouraged young people from smoking, and health professionals explained why smoking is dangerous to health. Inconsistency results were obtained for the factor: frequency of antismoking messages seen on billboards. While there was no difference in the odds of reported smoking between those who had not seen messages on billboards and those who had seen a lot of messages, respondents who had seen a few messages were more likely to report smoking compared to those who had not seen any message.

**Table 2:** Results of antismoking messages and smoking status in multivariate analysis, adjusted for age and sex in the Somaliland GYTS 2004

Factor	Adjusted Odds ratio (95% confidence interval)
Frequency of antismoking media messages seen or heard	
A lot	0.68 (0.55, 0.84)
A few	0.75 (0.58, 0.96)
None	1
Family member discussed harmful effects of smoking	
Yes	0.61 (0.52, 0.71)
No	
Frequency of antismoking messages seen at sports events, fairs, concerts, community events or social gathering	
A lot	1.30 (1.06, 1.50)
Some	0.90 (0.72, 1.13)
None	1
Taught dangers of smoking	
Yes	0.90 (0.72, 1.13)
No	1
Taught that smoking makes teeth yellow, causes wrinkles and smokers smell badly	
Yes	0.62 (0.52, 0.74)
No	1
Frequency of antismoking posters seen	
A lot	0.93 (0.73, 1.18)
Some	1.06 (0.83, 1.36)
No	1
Frequency of antismoking messages seen on billboards	
A lot	0.87 (0.66, 1.14)
Some	2.34 (1.86, 2.94)
No	1
Frequency of antismoking messages seen in newspapers or magazines	
A lot	1.17 (0.92, 1.48)
Some	0.82 (0.67, 1.00)
No	1
Taught that most people of respondent's age do not smoke cigarettes	
Yes	0.81 (0.69, 0.95)
No	1
Health professional explained why smoking is dangerous to health	
Yes	0.97 (0.83, 1.14)
No	1
Religious organization discouraged young people from smoking	
Yes	0.70 (0.60, 0.82)
No	1
Youth groups discouraged young people from smoking	
Yes	0.89 (0.76, 1.04)
No	1

### Factors associated with smoking

As shown in Table 2, respondents who discussed harmful effects of smoking cigarettes with their family members were 39% (OR = 0.61, 95%CI 0.52, 0.71) less likely to report smoking compared to those who did not discuss with their family members. Compared to respondents who had not seen antismoking media messages, those who had seen a few or too many messages were 25% (OR = 0.75, 95%CI 0.58, 0.96) and 32% (OR = 0.68, 95%CI 0.55, 0.84) less likely to report smoking, respectively. Respondents who had seen too many antismoking messages at social gatherings were 1.30 (95%CI 1.06, 1.60) times more likely to report smoking compared to those who had not seen the antismoking message at social gatherings. Meanwhile, respondents who were taught that smoking makes teeth yellow, causes wrinkles and smokers smell badly were 38% (OR = 0.62, 95%CI 0.52, 0.74) less likely to report smoking compared to those who were not taught. Compared to respondents who were not taught that most people of the respondent's age do not smoke, those who were taught were 19% (OR = 0.81, 95%CI 0.69, 0.95) less likely to report smoking. Finally, respondents who reported that religious organizations discouraged young people from smoking were 30% (OR = 0.70, 95%CI 0.60, 0.82) less likely to report smoking compared to those who did not report the same.

## Discussion

The prevalence of current smoking was 15.8% with no sex difference in reported smoking prevalence between males and females. The effect of sex on susceptibility to smoking is unclear. Some studies found that boys were at increased risk, some studies found that it is girls who were at increased risk, while others found no significant difference males and females [14-19].

In multivariate analysis, some knowledge or information related factors were associated with reported non-smoking while others were associated with reported smoking. Factors associated with knowledge of the harmful effects of smoking (having discussed with family or taught in school about harms of smoking) were associated with reported non-smoking. According to the HBM, teaching adolescents on the dangers of smoking may be important to enhance their perception of the severity of the condition. Individuals who perceived smoking as harmful to health would be less likely to smoke. In addition, the KAP model's aim would be to improve the knowledge levels of the adolescents thereby leading to behavioral change. Previous reports on school based smoking intervention programs have shown mixed results [20-22]. We again believe that the content and mode of delivery of these programs do matter as to their effectiveness.

It is interesting to note that exposure to anti-smoking messages through health workers, and print media (posters, billboards, newspapers, and magazines) was not associated with current reported smoking status. However, having been exposed to anti-smoking messages through sports or community events seem to have achieved a negative result of exposure to these messages being associated with reported smoking. This finding highlights the fact that the content and the delivery mode of anti-smoking messages may result in possibly unintended consequences.

Cohen et al [23] in their review of 399 televised anti-smoking advertisements that were designed to discourage adult and teen smoking found that most advertisements had emphasized the consequences of smoking more than the viewer's self-efficacy to quit smoking. Within the HBM and KAP models, these advertisements may have improved knowledge of harmful effects of smoking (perceived severity) but were limited in promoting self-efficacy that may spur the smoker to quit. Furthermore, advertisements were more likely to use informational and humor appeals, rather than sadness, fear, or anger appeals. Biener et al [24] have reported that anti-smoking advertisements featuring messages about serious health consequences that had been independently rated as high in negative emotion were more likely to be recalled and were perceived as more effective by youth survey respondents than ads featuring messages about normative behavior for teens or ads relying on humor. Advertising intensity, while contributing to recall, was negatively related to perceived effectiveness.

Flay and Sobel [25], and Rogers and Storey [26] have reported that mass media anti-smoking messages that promote inter-personal communication are more effective than those that do not. We found that adolescents who were exposed to interpersonal communication were less likely to report smoking than if they had not. We also believe that many of the billboards, posters, and newspaper anti-smoking messages may have possibly been a health warning on a pro-tobacco advert. Such advertisements, promoting glamour are less likely to result in reduced smoking among adolescents. Thrasher et al [27] have reported that cigarette packages whose warning labels contain prominent graphic imagery are more likely than text-only warning labels to promote smoking-related knowledge and smoking cessation.

Mass media campaigns are performed to promote health knowledge, change attitudes and may change behavior [28-32]. The KAP model, if effective, would be expected to lead to reduced smoking rates among adolescents. Staten and Ridner [33] have reported that inappropriate messaging may be less effective if no targeting is used.

We found that adolescents who reported that their religion discouraged smoking were less likely to report smoking. The lower rates of reported smoking could have been mediated through social norms within society where if society perceives a practice as unbecoming, adolescents are less likely to smoke. However, results on the positive role of religion are not consistent. Knowledge of pronouncement that smoking was sin was not associated with lower level of smoking in Egypt [34].

Adolescents who were taught that people of their age do not smoke may have reported less smoking as a result of improved efficacy that they are able not to smoke, as well as in conformity to normative beliefs (social cognitive theory).

### Limitations of the study

This study has a number of limitations. Firstly, data were collected through self-reports. To the extent that study participants misreported, either intentionally or inadvertently, our results may be biased. The study also only recruited students. Adolescents who were out of school were, therefore, not represented. Furthermore reported current cigarette smoking was not verified by appropriate biomarkers such as cotinine level or exhaled carbon monoxide [35-37].

## Conclusion

In a study of school-going adolescents in Somaliland, we found that adolescents who were exposed to anti-tobacco messages or discussions in school and at home were less likely to report smoking than those who were not exposed to such experiences. A combination of school and home based antismoking interventions may be effective in the control of adolescent smoking in Somaliland. Exposure to anti-smoking print messages was not associated with reported non-smoking in the current study, and we propose that studies be done on the effectiveness of anti-smoking messages (in terms of size, color and content) on adolescent smoking in Somaliland.

## Competing interests

The authors declare that they have no competing interests.

## Authors' contributions

SS led data analysis, participated in the interpretation of findings and drafting of manuscript. ASM participated in the interpretation of the findings and drafting of the manuscript. ER participated in the interpretation of the findings and drafting of the manuscript. All authors read and approved the final manuscript
